# Medical Ethics

**Published:** 1894-06-30

**Authors:** 


### Editor's Letter-Box.

[Our correspondents are reminded that proxility is a great bar to publication, and that brevity of style and conciseness of statement greatly facilitate early insertion.]

### MEDICAL ETHICS.

"A Correspondent" writes: In a small town in North Wales a young medical man recently started practice. He did not buy one, but simply put up his plate. He is a Nonconformist and was naturally supported by his Church. Nonconformity is very strong in the neighbourhood. An old medical practitioner who is a Churchman holds several public appointments, among which is that of medical officer for a parish which is really part of the town, the town being divided into two parishes, the one parish lying on one side of the river and the other on the opposite side of the river. The old doctor has a residence that side of the river opposite to where he holds the parish appointment, but he has a qualified assistant who lives in the parish, and he also has a surgery there. On account of his non-residence in the parish the appointment was made annually, but a few days ago he was to have been re-appointed. It was thought, as on previous occasions, this would have been unanimous and only of a formal character. The Nonconformists planned to supplant the old practitioner by electing the young doctor to the position. Whether the new comer approached certain of the Guardians on the subject, or whether they approached him, it does not matter, but undoubtedly he countenanced and encouraged all the proceedings. He attended the Board-room six miles from his residence and canvassed, urging his claims on the fact of his being a co-religionist of certain members of the Board; and he misrepresented the facts of the case by stating that the old practitioner would have all his forces together, and would have as many of the ex-officios as he could possibly muster. This was not the case, as the old medical officer, as before mentioned, understood the appointment would be unanimous, and took no steps in the matter; in fact, he was at the time on holiday, and neither he nor his assistant knew anything about the affair until the election was over. The young doctor did not live in the parish in question, but he has since taken a house which belongs to one of the lady Guardians, whose brother is an ex-officio Guardian and championed his cause. From the newspaper reports it will be seen that against the present parish doctor there was an entire absence of any charge of neglect or a single imputation which reflected discredit on him; yet the young practitioner was appointed practically by the votes of ex-officio Guardians, who but rarely favour the Board with their judicious counsels at other times. No other doctor had an opportunity of applying for the post, which, indeed, had not been publicly declared vacant, the private agenda for the meeting merely stating the re-appointment of the old practitioner as medical officer of the parish in question. Is not this a grave breach of professional etiquette? Were the Guardians acting within the terms of the Local Government Acts by electing a man when no previous notice of motion had been given of bringing one forward, neither had there been any intimation given to the present medical officer?

Would it be considered right for one doctor to call uninvited at the house of another doctor's patient who was lying ill and press to see the patient? This has been done, and the most ample proofs are forthcoming.

Would it be considered right for a doctor to push his way into people's houses, and on the plea that they are of the same religious persuasion, press upon them his claims where they have regular doctors and are thoroughly well pleased with them? This is another way that this young man has of getting a practice together.

